# Complete Genome and Methylome Sequences of *Salmonella enterica* subsp. *enterica* Serovar Panama (ATCC 7378) and *Salmonella enterica* subsp. *enterica* Serovar Sloterdijk (ATCC 15791)

**DOI:** 10.1128/genomeA.00133-16

**Published:** 2016-03-17

**Authors:** Kuan Yao, Tim Muruvanda, Richard J. Roberts, Justin Payne, Marc W. Allard, Maria Hoffmann

**Affiliations:** a Division of Microbiology, Office of Regulatory Science, Center for Food Safety and Nutrition, U.S. Food and Drug Administration, College Park, Maryland, USA; b School of Systems Biology, George Mason University, Manassas, Virginia, USA; c New England Biolabs, Inc., Ipswich, Massachusetts, USA

## Abstract

*Salmonella enterica* spp. are pathogenic bacteria commonly associated with food-borne outbreaks in human and animals. *Salmonella enterica* spp. are characterized into more than 2,500 different serotypes, which makes epidemiological surveillance and outbreak control more difficult. In this report, we announce the first complete genome and methylome sequences from two *Salmonella* type strains associated with food-borne outbreaks, *Salmonella enterica* subsp. *enterica* serovar Panama (ATCC 7378) and *Salmonella enterica* subsp. *enterica* serovar Sloterdijk (ATCC 15791).

## GENOME ANNOUNCEMENT

*Salmonella enterica* subsp. *enterica* serovar Panama was first isolated in the course of an investigation of a food-borne infection among soldiers stationed in Panama in 1934 (1). Since then, *S.* Panama has been isolated from food, animals, and water. It belongs to the serogroup D1 and causes gastroenteritis in humans (2). Importantly, this serotype tends to cause invasive diseases such as bacteremia and meningitis in children (3). The infections are usually obtained through the ingestion of contaminated food and can also be acquired from the consumption of contaminated breast milk (2).

*Salmonella enterica* subsp. *enterica* serovar Sloterdijk was first identified in 1964 (4). It was isolated from a family outbreak of salmonellosis in the Netherlands. Though *S.* Sloterdijk is not commonly found in the United States, it has been detected in raw oysters using PCR amplifications (5).

We received two clinical type strains from the American Type Culture Collection (ATCC, Manassas, VA, USA). The clinical strain of *S.* Panama (ATCC 7378) was isolated from an infant in New York, while *S.* Sloterdijk (ATCC 15791) was isolated from a family outbreak of salmonellosis in the Netherlands. Both isolates were cultured in Trypticase soy broth (Becton, Dickinson, Franklin Lakes, NJ, USA) overnight at 37°C. The genomic DNA was isolated from the overnight cultures using the DNeasy blood and tissue kit (Qiagen, Inc., Valencia, CA, USA). The DNA was sequenced using the Pacific Biosciences (PacBio) RS II sequencing platform, as previously reported (6, 7). Genomic DNA was sheared into approximately 20-kb fragments using g-TUBE (Covaris, Inc., Woburn, MA, USA). The library was prepared based on the 20-kb PacBio sample preparation protocol and sequenced using P6/C4 chemistry on four single-molecule real-time (SMRT) cells with a 240-min collection time. The continuous long-read data were *de novo* assembled using the PacBio hierarchical genome assembly process (HGAP version 3.0) with default parameters (8). The assembled sequences were annotated using the NCBI Prokaryotic Genome Annotation Pipeline and subsequently deposited at DDBJ/EMBL/GenBank.

The closed *S.* Panama genome sequence was sequenced with 127× coverage. The complete genome size was 4,555,576 bp with a G/C content of 52.28% and consisted of 4,387 genes. Using PHAST (9) analysis we identified one intact prophage, Salmon-RE-2010. The *S.* Sloterdijk genome sequence was fully closed with 77× coverage and has a genome size of 4,817,791 bp with a G/C content of 52.20%. The complete *S.* Sloterdijk genome contained 4,633 genes. PHAST analysis identified one intact prophage, Gifsy-2.

Using the PacBio RS II sequencing platform, the kinetic variations of nucleotide incorporation rates to infer DNA methyltransferase activities was detected (10). The SMRT data of the methylomes were analyzed and are summarized in Table 1. They are also deposited in REBASE (11) and can be found for *S.* Panama at http://rebase.neb.com/cgi-bin/pacbioget?16672 and for *S.* Sloterdijk at http://rebase.neb.com/cgi-bin/pacbioget?16673.

**TABLE 1:** Summary of active methylases and their recognition sequences

Strain	Assignment	Methyltransferase specificity^a^	Methylation type	Restriction modification type
*S.* Panama	M.Sen7378I	CAG**A**G	m6A	III
	M.Sen7378II	A**T**GC**A**T	m6A	II
	M.Sen7378ORF5420P	G**AT**C^b^	m6A	II
	M.Sen7378DamP	G**AT**C^b^	m6A	Orphan
*S.* Sloterdijk	M.Sen15791I	CAG**A**G	m6A	III
	M.Sen15791III	A**T**GC**A**T	m6A	II
	M.Sen15791Dam	G**AT**C	m6A	Orphan
	M.Sen15791II	CG**A**NNNNNN**T**RCC	m6A	I

^a^ The methylated bases, all m6A, are indicated by a boldface “A” if they are on the strand shown or a boldface “T” if they are on the complementary strand.

^b^ GATC cannot be assigned unambiguously, but it is likely that M.Sen7378DamP is active.

### Nucleotide sequence accession numbers.

The complete genome sequence of *S.* Panama is available in GenBank under the accession number CP012346. The complete genome sequence of *S.* Sloterdijk is available in GenBank under the accession number CP012349.
